# Collective Investigation of the Influence of the Silver Nitrate Injections on Phthisis

**Published:** 1901-09

**Authors:** Thomas J. Mays

**Affiliations:** 1829 Spruce St., Philadelphia, Pa.


### CORRESPONDENCE.

### COLLECTIVE INVESTIGATION OF THE INFLUENCE OF THE SILVER NITRATE INJECTIONS ON PHTHISIS.

*To the Members of the Medical Profession:*

In 1892 the undersigned began a collective investigation of the action of cold in the treatment of acute pneumonia, and there is reason for believing that this procedure, which resulted in gathering four hundred cases of this disease thus treated, with a death-rate not quite five per cent., was an important factor in calling attention to the utility of that treatment, and in introducing it to the profession of this country. That research was based on the conviction that no remedy can be called truly successful until it has passed the exacting crucible of clinical experience, and it is now proposed to apply the same ordeal to the silver-injection treatment of phthisis, which, in a large hospital, dispensary and private practice, reaching over a period of three years, and during which many thousand injections were administered, has given me greater satisfaction than any other method that I have ever employed. In keeping with the above expressed feeling, a cordial invitation is herewith extended to those members of the profession who have the inclination and opportunity to investigate this method of treating phthisis and to whom a reprint on the subject, with full information and blanks to report cases, will be cheerfully sent on application.

THOMAS J. MAYS, M.D.

1829 Spruce St., Philadelphia, Pa. Aug. 15, 1901.

### A LEAGUE AGAINST SYPHILIS.

As an outcome of the Congress on the Prophylaxis of Syphilis held in Brussels, in 1899, a league against syphilis has recently been founded in Paris. The president is Professor Fournier; the vice-presidents are Drs. Bérenger, Brissaud, and le Pileur. Dr. Barthélemy is secretary-general.

